# An overview of Iran's actions in response to the COVID-19 pandemic and in building health system resilience

**DOI:** 10.3389/fpubh.2023.1073259

**Published:** 2023-02-01

**Authors:** Mohammad-Mehdi Gouya, Katayoun Seif-Farahi, Payman Hemmati

**Affiliations:** ^1^Department of Infectious Diseases, Iran University of Medical Sciences and Health Services, Tehran, Iran; ^2^Iranian Center for Communicable Diseases Control, Ministry of Health and Medical Education, Tehran, Iran

**Keywords:** Iran, health system, resilience, COVID-19, health emergencies, equity, pandemic, primary health care (PHC)

## Abstract

This article is part of the Research Topic ‘Health Systems Recovery in the Context of COVID-19 and Protracted Conflict'. The considerable human, social, and economic impacts of COVID-19 have demonstrated a global lack of health system resilience, highlighting gaps in health system capacities due to fragmented approaches to health system financing, planning, and implementation. One of the key actions for ensuring equitable essential health services in all countries in normal situations as well as emergencies is through strengthening the primary healthcare (PHC) system. In the context of the unfolding pandemic, the Iranian Ministry of Health and Medical Education (MoHME) undertook a variety of strategic actions to ensure the sustainability of health services during the current health emergency and to promote health system resilience against future shocks. Right after the Alma-Ata declaration in 1978, MoHME pursued the PHC philosophy incorporating the principles within the WHO health system framework and its six building blocks. In response to the evolving pandemic, MoHME put in place several interventions to ensure the maintenance of essential health services in addition to the provision of response. Some interventions were new, informed by global experience with COVID-19, while others leveraged existing strengths within the existing health system. Those were taking a whole-of-government approach; leveraging the PHC capacity; supporting the workforce; strengthening preparedness and response; improving access to medicines, vaccines, and health products; and leveraging the health information system into the pandemic response. Health system strengthening that promotes resilience is imperative for governments as health systems are fundamental to sustainable socioeconomic development. In recognition of this, the WHO Eastern Mediterranean Regional Office (EMRO) has recently outlined regional priorities for advancing universal health coverage (UHC) and ensuring health security. Iran's approach both prior to and during the pandemic is strongly aligned with those regional priorities, which are “*primary health care-oriented models; enhancing health workforce; promoting equity; enabling environment for research; improving access to countermeasures; and fostering health system resilience.”*

## Introduction

The considerable human, social, and economic impacts of COVID-19 have demonstrated a global lack of health system resilience, highlighting gaps in health system capacities due to fragmented approaches to health system investment, planning, and implementation. Widespread and prolonged disruptions to essential health services were seen in virtually all countries, regardless of their income status or their level of development (1). It has been demonstrated that reducing both individual and population vulnerability to health threats is a key factor in ensuring sustainable economic development (2). There is global recognition of the need to strengthen health systems to support universal health coverage (UHC) and health security in order to build resilience against future public health emergencies (PHEs) (3). One of the key actions for ensuring equitable essential health services in all countries in normal situations as well as emergencies is through strengthening the primary healthcare (PHC) system. This requires governance, advocacy, planning, and financing at national, regional, and global levels. The Islamic Republic of Iran (IR Iran) has pursued the PHC approach since 1979, shortly after the Alma-Ata Declaration was adopted in 1978 (4).^1^ The country has conceptualized its approach to strengthening primary healthcare in terms of the WHO health system framework and its six building blocks, i.e., leadership, financing, health workforce, medical products, vaccines, and technologies, service delivery, and health information system (5).

In the context of the unfolding pandemic, the Ministry of Health and Medical Education (MoHME) undertook a variety of strategic actions to respond to the pandemic, to ensure the sustainability of health services during the current health emergency, and to promote health system resilience against future shocks.

## Context

Iran is the seventeenth largest country in the world with an area of 1.648 million square kilometers (6), with diverse climatic conditions, vegetation, and animal species, and a variety of ethnicities, languages, cultural practices, and levels of socioeconomic advancement. Consequently, different provinces in the country are facing diverse environmental and socioeconomic conditions. The country's population is ~86 million people (7) of which about 61 million live in urban areas.

### Overview of Iran's health system prior to the pandemic

**Figure 1:**
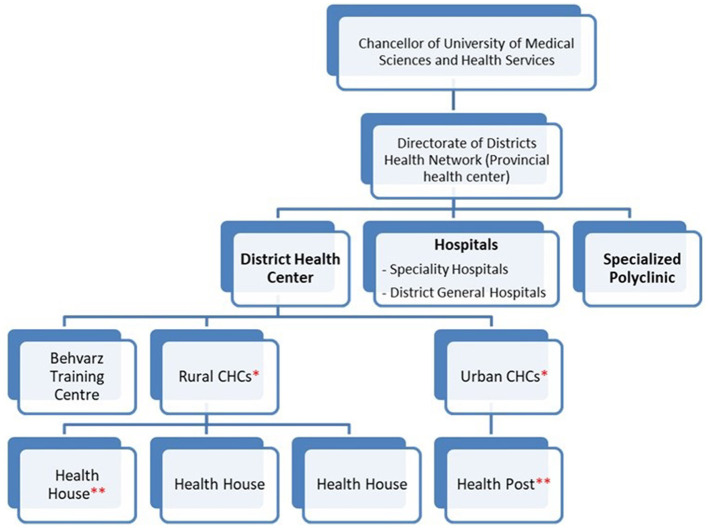
The structure of Iran's health system in each province. *Each rural CHC covers almost 8,000 people, whereas an Urban CHC covers ~37,500 people. **Each Health House serves almost 1,000 rural people, while each Health Post provides services for 12,500 city dwellers.

The health system falls under the mandates of the MoHME in Iran which is responsible for the provision of health services as well as medical education, research, and health policy (8). Iran has a long history of investment in PHC, which began immediately after the Alma-Ata declaration in 1979. Since its establishment, there has been a steady improvement in health indicators (9). The system has expanded countrywide with more than 500 district health centers and 4,600 rural and urban health centers titled “comprehensive health care centers (CHC),” which are present even in remote rural areas. There are ~18,000 community-based “health houses” in rural areas where community health workers (Behvarz) provide essential health services as well as referral for diagnosis and treatment of public health issues to higher levels, i.e., CHCs where required (refer to Figure 1). There are ~5,000 health posts in urban areas countrywide. Each urban CHC covers a population of 37,500 and each health post, which is affiliated to an urban CHC, covers 12,500 people, whereas a rural CHC covers a population of 8,000 and every affiliated health house covers 1,000 people. Service delivery intends to provide quality interventions through the PHC system. As of 2005, MoHME established the “Family Physician Program” in rural areas, which introduced the role of the family physician, with the latter providing public health, diagnosis, and treatment services. This has been helpful in decreasing the burden on hospitals by treating patients at the early stages of illness. There are ongoing efforts to expand the program in urban areas (10).

There are more than 5 million non-Iranian nationals (asylum seekers, refugees, and migrants) currently living in Iran. This is an enormous challenge for the country's health system and one, which Iran has taken seriously and systematically. The country has an established refugee health extension program through which refugee-specific CHCs (Behdasht-Sara) are constructed within refugee camps. Peer physicians and community health workers (Behbakhsh) from the refugee population, who have received training in Iran, are recruited to provide services in the camps. The United Nations High Commissioner for Refugees (UNHCR) supports the construction of specific CHCs and the recruitment of those according to the health extension program. Each refugee-specific CHC is affiliated with the nearest CHC. In addition, if a refugee attends any urban or rural CHC, they are entitled to receive the same level of public health services at no cost as Iranian residents. Peer health workers selected from the migrant population are recruited in health houses in refugee/migrant camps and serve as assistants of health staff in the PHC system. Some of these health houses are established and recruited through UNHCR support as an international partner; however, given the expanding refugee population, demand is greater than the current capacity.

As of 2017, there were 981 hospitals with 129,604 ordinary beds and 8,264 adult Intensive Care Unit (ICU) beds in the country. The hospital bed-population ratio was lower compared with developed countries, and their distribution varied by geographical area.

While there are two sectors for the delivery of clinical services within Iran, i.e., public and private, all public health services such as immunization, children and maternal health, mental health, etc., are provided free of charge. In the public sector, 70% of treatment service fees are covered by the national health insurance system. The MoHME operates public hospitals, both general and special ones, throughout Iran. Public hospitals are typically under the direct management of universities, while private hospitals are regulated and supervised by MoHME.

The national electronic health record (EHR) system has been operating since 2007 following the Open-EHR reference model. For each patient or family who comes to CHCs, health posts and houses in urban and rural areas an EHR^2^ is filed. Each family member/or patient has their own EHR. For approximately 65% of people, their EHRs are linked to the EHRs of their first degree relatives. After the COVID-19 vaccination campaign, the share of Iran's population has been registered in the national EHR system reached over 90%. There are four different EHR systems countrywide. More than 800 public hospitals transfer inpatient data to the EHR infrastructure every day.

The Iranian integrated disease surveillance and response system (IDSR) was established in 2016. Although called a “syndromic surveillance system” (Triple S or SSS) with its original early warning (EWAR) component based on a syndromic approach, Triple S is a broad-based four-module system including modules on early warning, case-based epidemiological surveillance, lab-based surveillance, and automated reports (11). It was developed with WHO support. The study phase was started in 2011 and concluded with the first version of the electronic platform in 2016. Currently, a variety of IT solutions are offered to incorporate SSS in the EHR system as a built-in module, which is an effort to integrate SSS with evolving EHR platforms. The SSS early warning component is based on 17 syndromes and their minimum datasets, which is the country's EWAR mechanism for health emergencies. Currently, the system has integrated ~40 communicable diseases of acute respiratory and food-and-water-borne nature under acute respiratory and acute diarrhea syndromes.

### The COVID-19 pandemic period

Iran was one of the first countries to experience the rapid progression of the COVID-19 pandemic. The first peak occurred at the end of March 2020, with around 3,200 daily cases, which created a very challenging situation for the people and officials (4).

Iran faced constraints in relation to personal protective equipment as well as hospital and laboratory equipment as a result of the global supply shortage due to the rapid and high demand exacerbated by delayed delivery timeframes at the beginning of the pandemic. Restrictions on the international transfer of payments from Iran's banking system to international banks added to this challenge.

## Key interventions in Iran's response to the COVID-19 pandemic

In response to the evolving pandemic, Iran put in place several interventions to ensure the maintenance of essential health services in addition to the provision of response. Some interventions were new, informed by global experience with COVID-19 while others leveraged existing strengths within the existing health system.

### Whole-of-government approach

In response to the pandemic, the government established several mechanisms to support a whole-of-government, whole-of-society approach including the National Steering Committee (NSC) of COVID-19. This committee is led by the President and as of late summer 2022, this committee continues to meet, each Saturday, with different cabinet ministers and high-ranking national authorities (Box 1).

Box 1 **Illustrative example of ministries and sectors represented in the National Steering Committee.**

**Table:** 

• The President	• The Judiciary System of I.R. Iran
• Ministry of Interior	• National Security Council
• Ministry of Health and Medical Education	• Central Bank of Iran (CBI)
• Ministry of Cooperatives, Labour and Social Welfare	• The Islamic Consultative Assembly (Parliament)
• Ministry of Economic Affairs and Finance	• Police
• Ministry of Culture and Islamic Guidance	• I.R. Iran Medical Council
• Ministry of Foreign Affairs	
• Ministry of Education	
• Ministry of Industry, Mine and Trade	
• Ministry of Science, Research and Technology	
• Ministry of Petroleum • Ministry of Roads and Urban Development	
• Ministry of Information and Communications Technology (ICT)	

Another whole-of-government mechanism called the National Committee for COVID-19 management has been established by the Ministry of Interior, which is in charge of the implementation of policies set by the NSC. All the executive officials of the counterpart ministries of the NSC are members of this committee.

As of late summer 2022, when the seventh wave of the pandemic had just ended, provincial health authorities were continuing to hold weekly virtual meetings with health authorities at the national level to review the pandemic situation, discuss the challenges, exchange lessons learned, and address any issues identified (2).

### Leveraging the PHC capacity

Before COVID-19, Iran had invested heavily in PHC with the current flagship program. “Each home acts like one health post”^3^ being rolled out, with a focus on systematically strengthening PHC in terms of disease prevention and health promotion (7). From the very beginning of the COVID-19 pandemic, this existing PHC capacity was leveraged with the MoHME designating some CHCs as selected 16-h COVID-19 centers in each district. Routine health services continued to be offered in the other CHCs as a mechanism to achieve a more resilient PHC system. This mechanism helped to maintain essential services (UHC) while necessary care was given to mild cases of COVID-19 in selected centers as part of the response to a health emergency. This strategy also follows the principle of infection and prevention control in health facilities. MoHME designated ~1,050 COVID-19-selected health centers among 4,600 CHCs countrywide. Those designated centers have been operating during successive pandemic waves and have had a significant impact in reducing the burden on hospitals as a load of patients presenting for a consultation to those PHC centers was almost 10 times more than those presenting to hospitals. This difference demonstrates the potential of the PHC system to safeguard secondary and tertiary care and has made the hospitals more resilient to this burdensome pandemic. All selected COVID-19 centers and CHCs were active in contact tracing with the aid of military forces and volunteers.

Community-based nursing care centers were expanded during COVID-19 to deliver services at home to support the early discharge of patients from hospitals and to secure the continuity of care in the community. Standard packages of nursing services at home were developed for several priority conditions to standardize and systematize quality and harness cost-effective nursing services at home. While the expansion of community-based nursing care was welcome, it was also necessary to ensure the provision of other occupational categories including midwives, physiotherapists, clinical psychologists, and social workers in such centers. Thus, MoHME has made an effort to connect the centers for counseling and nursing care at home with CHCs with the aim of increasing access to these services while reducing the visits to emergency rooms, as well as reducing the health system's costs and hospital bed occupancy rate.

In order to protect elective and routine operations and treatment services, MoHME designated some hospitals for COVID-19 inpatients at the beginning of the pandemic. In addition, in the last year, the ministry has increased acute hospital bed capacity by 10,000 hospital beds, bringing the current total to 155,000 (12). During the recovery phase, these recent developments join the routine service capacities for the normal situation.

### Supporting the workforce

The Iranian health system has a mix of healthcare staff including doctors, nurses, midwives, nutritionists, mental health experts, dentists, environmental and occupational health workers, communicable disease experts, lab technicians, and scientists, which is well-distributed in the country. On average, there are ~1.6 physicians for every 1,000 population (13). According to the World Bank, the physician per population rate has been 1.4, 1.7, 2.6, and 4.9 in the Middle East and North Africa, the globe, North America, and European Union, respectively, in 2017.

The country's health system undertook several innovative approaches to expand the capacity of the workforce in response to COVID-19 including the use of military staff and volunteers in COVID-19 centers and CHCs, especially for active case finding and contact tracing of COVID-19 cases. Many post-graduate students (clinical residents) from other clinical disciplines received short-course training and were redeployed during the pandemic to provide assistance to infectious disease specialists, pulmonologists, internists, pediatricians, and ICU specialists who were on the frontline of providing care to patients with COVID-19. During the peak of the pandemic, a national database of nurses was established to enable the recruitment and redeployment of nurses across provinces.

Regarding surge capacity, the workforce has been strengthened by hiring through 90-day contracts and by extending the time of the contract. In addition to this, one of the measures taken by MoHME has been the periodic assessment of health system capacity in each province, especially during the peak of pandemic waves to support the redeployment of staff. For example, when the delta wave hit the country from July till the end of September 2021, the clinical sector of some provinces was overwhelmed, using the periodic assessment to identify workforce capacity. The Ministry was able to mobilize reserve physicians and nurses to the hospitals of severely affected provinces (surge capacity) to maintain clinical services for hospitalized patients.

Recognizing the serious risk of burnout among frontline healthcare workers (HCWs), Iran has followed a variety of actions to support and empower HCWs in the clinical sector including the development of a program for improving their psychosocial wellbeing, to address mental health issues and prevent burnout during COVID-19.

Looking to ensure future capacity, capacity-building for online teaching was addressed in schools of Medicine and Nursing since 2020, by equipping these with the necessary infrastructure, planning, and conducting training of trainers (TOTs) courses for faculty staff. Thus, it was possible to hold online classes for the students. In addition, many webinars were held during the pandemic to support the training of the healthcare workforce and exchange experiences and learning.

###  Strengthening preparedness and response through multisectoral and multidisciplinary working and partnerships with international agencies

In late June 2022, we conducted a multisectorial multidisciplinary workshop in Tabriz city, Iran, attended by 175 stakeholders of all relevant MoHME departments, ministries, and organizations to reach a consensus and consolidate an integrated Pandemic Influenza, COVID-19, preparedness, response, and recovery plan (IPICPRP), which has also integrated acute respiratory infections with epidemic potential. The attendance rate was high (just two invitees were absent). The three back-to-back multidisciplinary workshops helped gather perspectives and feedback on the draft plan, which followed WHO recommendations (14, 15).

The updated pandemic plan was developed in light of the COVID-19 response. In the plan, strategic actions are categorized according to four phases (inter-pandemic, alert, pandemic, and recovery phases), and respective activities for each action with timelines, responsible agency, partners, and budget are included with the aid of extensive multidisciplinary and multisectoral deliberations.

New strategies taken by the Global Fund and Gavi to improve service coverage and address inequities in Iran demonstrate how the partnership of international agencies can improve some building blocks of the health system and secure health system functions including better access to health commodities and integrated communicable disease surveillance. Recent reclassification as a lower-middle income country (LMIC) by the World Bank will enable Iran to benefit from Gavi support as a beneficiary of COVID-19 and other vaccines. Gavi supports Advance Market Commitment (AMC) countries, which are mostly LMIC ones. This is important to combat pandemics and epidemics and can empower the health system of many countries against health emergencies. Iran received more than 10 million doses of COVID-19 vaccines from Gavi through the COVAX mechanism as a non-AMC country. However, as of 18 December 2022, the total vaccine shots received by the Iranian population reached approximately 155 million doses,^4^ which MoHME supplied mostly *via* bilateral contracts and domestic production.

During the pandemic, the Global Fund supported the COVID-19 response by providing personal protective equipments (PPEs), RT-PCR machines, and cold-chain equipment in support of COVID-19 vaccine deployment. After the pandemic, such equipment can continue to be leveraged to support health system infrastructures and improve service delivery, especially surveillance and response functions. Therefore, these partnerships are important in achieving a more resilient health system (16). Also, the Global Fund and Gavi have built effective mechanisms for market shaping, procurement, and supply chains of vaccines and health commodities, with the aid of the UNICEF supply division to deploy COVID-19 vaccines to many countries including Iran. This mechanism can leverage to procure affordable quality health commodities for the next health emergencies.

### Improving access to medicines, vaccines, and health products

COVID-19 medications and vaccines were distributed all over the country based on population size and provincial needs. The MoHME leveraged the already existing supply chain of the PHC system as shown in Figure 1. The first batch of COVID-19 vaccines became available in Iran in March 2021 and was distributed following a prioritization system that in the initial phase targeted frontline HCWs combating COVID-19, high-risk people in long-term care including the disabled and the elderly as well as veterans who had been affected by chemical weapons during the Iran–Iraq war in the 1980s and highly-disabled veterans residing at home.

As sufficient vaccines became available in the summer of 2021, vaccination was expanded to include all people regardless of nationality, religion, or ethnicity. Leveraging the existing countrywide PHC including its vaccine-distribution capacity, the Iranian MoHME established roughly 1,200 mass vaccination centers in stadiums, museums, schools, and so forth as an adjunct to PHC. This enabled Iran to achieve a COVID-19 primary vaccination coverage rate of around 75% up until January 2022.

In addition, Iran has developed a local production capacity for seven domestic COVID-19 vaccines, which can be regarded as one of the key lessons from the pandemic and has established the country's capacity for the production of medical countermeasures for epidemic-prone diseases. These include vaccines across different technology platforms such as protein-based, inactivated, and adenoviral vector vaccines. Local production of an mRNA-based vaccine is currently under investigation (December 2022). Efforts are ongoing to receive the WHO emergency use listing (EUL) of the locally produced vaccines with the support of Iran's Food and Drug Administration (FDA). Many clinical trials were designed and implemented in different phases on locally produced vaccines^5^ (17, 18) by Iranian research institutes and Clinical Research Organizations (CROs) during COVID-19 under the supervision and support of Iran's FDA, National Committee for COVID-19 Vaccine, and the Undersecretary for Research and Technology of MoHME.

The current capacity can be leveraged to produce other vaccines, improve response capacities for other communicable diseases, and achieve a more resilient health system against the next health emergency. However, more support from WHO and WHO/EMRO is needed for technology transfer and assisting local producers in receiving WHO/EULs and prequalification in order to leverage currently existing capacities.

Some medications for treating COVID-19 were also produced locally, and Iran produced PPE locally to overcome the shortages, which were apparent at the beginning of the pandemic.

### Health information system

The Electronic Health Record (EHR), which was in place several years before the pandemic, was adapted to register suspected cases of COVID-19 and their contacts and to record delivered services including testing and treatments. Leveraging the existing EHR capacity, an electronic registry was added to support the national vaccination campaign against COVID-19.

The syndromic surveillance system (SSS) platform was adapted by adding COVID-19 under respiratory syndromes, i.e., ILI (influenza-like illness), SARI (Severe Acute Respiratory infection), and ARI (acute respiratory infection) in addition to the differentials of acute respiratory illness previously included such as Influenza types and subtypes.

## Discussion

Health system strengthening that promotes resilience is imperative for governments as resilient health systems are fundamental to improving, achieving, and maintaining equity in populations' health and wellbeing; responding to public health emergencies, and enabling sustainable socioeconomic development. In recognition of this, the 69th regional committee of WHO/EMRO published a technical article in September 2022 outlining regional priorities for advancing UHC and ensuring health security (preventing and controlling future health emergencies in the Eastern Mediterranean Region) by building health system resilience. These priorities include:

- Establishing primary healthcare-oriented models of care.
- Enhancing fit-for-purpose, fit-to-practice health workforce.
- Promoting equity and enhancing financial protection.
- Enabling an environment for research, innovation, and learning.
- Improving access to medicines, vaccines, and health products.
- Fostering an integrated approach in policy, planning, and investments for long-term health system resilience.

Iran's approach both prior to and during the pandemic is strongly aligned with these regional priorities. For example, the IPICPRP promotes an integrated approach in preparedness, response, and recovery planning for long-term resilience while strengthening health and emergency and disaster risk management by creating platforms to tackle multi-hazards. The multisectoral national committees established to support the COVID-19 response represent a whole-of-government approach to health that strengthens emergency management. Such structures can be maintained within the recovery phase to provide a holistic government approach to health, not just during emergencies. This multisectoralism was also apparent in the development of local capacities for the delivery of PPE, medicines, and vaccines and is aligned with improving access to medicines and technologies and securing supply chains in support of emergency preparedness and response. The multiple innovative approaches applied to ensure workforce capacities including redeployment and additional training are aligned with a fit-for-practice workforce.

By leveraging existing PHC capacities and infrastructure, Iran has been able to achieve, as of November 2022, a first-dose vaccination coverage of 73.8%. This rivals the figures for many developed countries such as Germany, France, and the USA with vaccination coverage of 77.74%, 80.51%, and 80.43%, respectively (19). With the aid of the PHC approach and the peer vaccination program for vulnerable populations, vaccine uptake in first- and second-doses is estimated to reach 91% and 68%, respectively, among the eligible refugees for vaccination. The targeted refugee population was approximately 4.3 million, which included both registered refugees and undocumented migrants. A total of 2.7 out of 4.3 million were older than 12 years, thus, they were considered eligible for immunization against COVID-19. Undocumented migrants were disproportionately affected by COVID-19 globally due to a combination of socioeconomic and cultural vulnerabilities, a population recognized as particularly hard to reach globally (20, 21). The high COVID-19 vaccination coverage among refugees and migrants in Iran was possible because of the pre-existing foundation of trust between this often hard-to-reach population and existing services. Trust has been identified as a key to effective emergency response.

The MoHME is currently planning to further build on this in support of health systems resilience by applying the approach to vulnerable populations pursued with COVID-19 vaccination to routine immunization. This will involve integrating routine immunization of refugee populations to fill the gaps in TB detection rate and COVID-19 vaccination.

Existing capacities in PHC and HIS were also leveraged to support the maintenance of services and to promote high uptake of COVID-19 vaccination. Disruptions to essential services were seen globally according to the 3rd report of pulse surveys, with an average decrease of 36% and 49% in communicable disease services and immunization coverage worldwide, respectively (1). In Iran, there was a 28% decrease in the TB detection rate during the COVID-19 pandemic and no drop in routine vaccination coverage in 2020–2021 (22).

During the COVID-19 peak, a guideline issued by the MoHME states that in case of a regular bed occupancy rate beyond 50% and ICU bed occupancy beyond 60%, the admission of patients for elective operations could be canceled by the chancellor board of the university. In the recovery phase of the pandemic, those beds have returned to routine healthcare services, e.g., devotion to elective procedures and operations (23).

According to World Bank data, Iran's GDP per capita stood at 2,756.7 in 2020 far below that recorded for Germany, France, and the USA which reached 50,801.8, 43,518.5, and 69,287.5 respectively (24). When placed in the context of the economic realities facing Iran, the approach taken demonstrates that it is not the absolute availability of resources but political will and the leveraging of all investments, including those focused on response efforts to strengthen health system foundations with PHC that builds resilient health systems. This approach ensures that investments yield long-term dividends and provides an example of the value of investing in PHC in routine times in support of health security during emergencies.

In spite of efforts made to integrate between different health information systems such as different EHR systems and surveillance platforms, e.g., SSS, the COVID-19 pandemic demonstrated that further work is needed in the area of integrated and interoperable health information systems to address the issue of data fragmentation.

Regarding technology transfer and local vaccine production, this is a cumbersome area that needs much more support from WHO and investment from global partners. Challenges in the electronic wiring between Iranian and international banking systems to supply medical countermeasures and support technology transfer is another constraint, which negatively affects health system resilience, and those need to be addressed before the hit of the next health emergency or pandemic.

## Strengths and limitations

The immense variety of strategic actions and activities taken by Iran in responding to the COVID-19 pandemic means that the picture we have drawn here is, inevitably, incomplete. Some of the omissions concern major initiatives, for instance, the establishment of a laboratory network of more than 500 labs with molecular diagnostics and/or viral sequencing capacities. The authors' own involvement in certain aspects of the COVID-19 response and not others is also likely to have biased the representation. Nonetheless, the article prioritized and synthesized a vast amount of evidence and experience of policy and decision-makers and academics from Iran's MoHME and leading Iranian universities and institutes. It can suggest ideas and lessons for other countries in responding to and recovering from future public health emergencies, particularly in demonstrating PHC-oriented models of care, mass vaccination centers, and building capacity in domestic vaccine production.

## Data availability statement

The original contributions presented in the study are included in the article/supplementary material, further inquiries can be directed to the corresponding author.

## Author contributions

All authors listed have made a substantial, direct, and intellectual contribution to the work and approved it for publication.

## Footnotes

^1^ https://en.wikipedia.org/wiki/Alma_Ata_Declaration

^2^ https://sib.behdasht.gov.ir/

^3^It means that one family member of a household can be linked to the community health worker of the neighborhood's health post and she/he can help advocate public health measures to her/his family members.

^4^ https://behdasht.gov.ir/

^5^Mostafavi E, Eybpoosh S, Karamouzian M, et al. Efficacy and safety of protein-based SARS-CoV2 vaccine (SOBERANA 02 and SOBERANA Plus): a double-blind randomized, placebo-controlled, phase 3 trial in Iran (2022).
